# Multi-Path Routing Algorithm for Wireless Sensor Network Based on Semi-Supervised Learning

**DOI:** 10.3390/s22197691

**Published:** 2022-10-10

**Authors:** Yiping Guo, Guyu Hu, Dongsheng Shao

**Affiliations:** 1 Command and Control Engineering College, People’s Liberation Army Engineering University, Nanjing 210007, China; 2 Unit 31106 of People’s Liberation Army, Nanjing 210007, China

**Keywords:** wireless sensor network, semi-supervised learning, evaluation, multi-path routing

## Abstract

Multi-path transmission can well solve the data transmission reliability problems and life cycle problems caused by single-path transmission. However, the accuracy of the routing scheme generated by the existing multi-path routing algorithms is difficult to guarantee. In order to improve the accuracy of the multi-path routing scheme, this paper innovatively proposes a multi-path routing algorithm for a wireless sensor network (WSN) based on the evaluation. First, we design and implement the real-time evaluation algorithm based on semi-supervised learning (RESL). We prove that RESL is better in evaluation time and evaluation accuracy through comparative experiments. Then, we combine RESL to design and implement the multi-path routing algorithm for wireless sensor networks based on semi-supervised learning (MRSSL). Then, we prove that MRSSL has advantages in improving the accuracy of the multi-path routing scheme through comparative experiments.

## 1. Introduction

Wireless sensor networks consist of a large number of sensor nodes. These sensor nodes use wireless communication to form a multi-hop self-organizing network system, so as to realize cooperative sensing, collection and processing of the information of sensing objects in the geographical area covered by the network [1].

**Figure 1:**
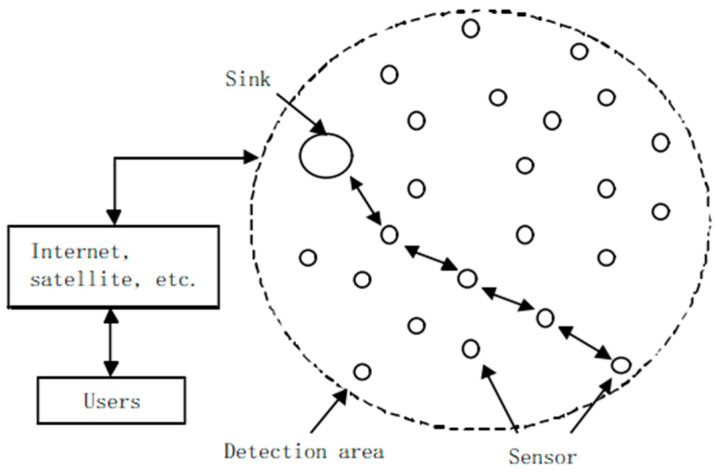
Wireless sensor network structure.

The structure of wireless sensor network is generally simple, as shown in Figure 1. The data transmission of the whole network is mainly the transmission process between the sink node and the source sensor node.

In the process of designing the traditional routing method of the wireless sensor network, the optimal path from the source sensor node to the sink node is generally used for data transmission. There are many problems with the traditional single-path transport mechanism. First, single-path transmission cannot guarantee the reliability of data transmission. Once the current path fails, the reliability of data transmission cannot be guaranteed because there is no backup path available. The rerouting caused by this consumes more (communication) energy, which is undoubtedly fatal to sensor nodes that are already short of energy. Second, single-path transmission puts more pressure on the network. If a large amount of data needs to be transmitted between the source sensor node and the sink node, blindly relying on a certain path for data transmission will make the energy consumption of each network node vary greatly. It often causes premature aging of some nodes [2], which eventually leads to a shortened network life cycle [3].

Multi-path transmission can well solve the data transmission reliability problems and life cycle problems caused by single-path transmission [4]. References [4,5] have proposed solutions to the problems caused by single-path transmission. Reference [5] proposed a graph routing algorithm with enhanced lifecycle (hereinafter referred to as QGRLE). The algorithm effectively alleviates the life cycle problem and considers the reliability of data transmission but does not effectively solve the problem of data transmission reliability. Reference [4] proposed a classical energy multi-path routing mechanism (hereafter referred to as CEMRM). The mechanism establishes multiple paths between the source node and the destination node and assigns a certain selection probability to each path according to the communication energy consumption of the nodes on the paths and the remaining energy of the nodes. This mechanism effectively alleviates life cycle issues and data transmission reliability issues. Reference [6] proposed a multi-path routing algorithm based on the genetic algorithm, which dynamically selects the optimal path with the minimum distance and minimum energy dissipation according to the cost function. Reference [7] proposed a multi-path routing algorithm based on quadratic programming. The algorithms proposed in references [6,7] can reduce energy consumption and routing overhead, but they are difficult to ensure the accuracy of the routing scheme.

Compared with single-path transmission, multi-path transmission has obvious advantages. However, the accuracy of the routing scheme generated by the existing multi-path routing algorithms is difficult to guarantee [8]. After the multi-path routing scheme is generated, the paths are selected in order for data transmission. Each additional selection is a waste of energy, and an inaccurate routing scheme will lead to a large amount of energy waste. The node energy in wireless sensor networks is limited, so the accuracy of the routing scheme is very important, but the existing schemes have not been deeply researched on this problem.

For the accuracy of the routing scheme, this paper considers using evaluation to improve the accuracy of the routing scheme. We evaluate the initial routing scheme immediately after it is generated and make adjustments to the schemes with abnormal evaluation (that is, the routing scheme is inaccurate), thereby effectively improving the accuracy of the final routing scheme. At present, the common algorithms used to evaluate routing schemes include fuzzy evaluation algorithm [9], adaptive evaluation algorithm [10] and so on.

Multi-path transmission can well solve the problems caused by single-path transmission. However, existing multi-path routing algorithms are difficult to ensure the accuracy of the generated routing scheme. The innovation of this paper is to solve the problem of low accuracy of multi-path routing schemes in wireless sensor networks (WSN) by means of evaluation.

The main research contents are as follows: (1) Section 2 covers the design and implementation of the real-time evaluation algorithm based on semi-supervised learning (RESL) with the goal of reducing evaluation time and improving evaluation accuracy. Then, we compare RESL with the two evaluation algorithms proposed in references [9,10]. The experimental results show that RESL is better in evaluation time and evaluation accuracy. (2) Section 3 combines RESL to design and implement the multi-path routing algorithm for wireless sensor networks based on semi-supervised learning (MRSSL). Then, we compare MRSSL with the two wireless sensor network multi-path routing algorithms proposed in references [4,5]. The experimental results show that MRSSL is better in the accuracy of the routing scheme.

## 2. Real-Time Evaluation Algorithm Based on Semi-Supervised Learning (RESL)

In this section, we design and implement the real-time evaluation algorithm based on semi-supervised learning (RESL). We verify the advantages of this algorithm in reducing evaluation time and improving evaluation accuracy through comparative experiments.

### 2.1. Algorithm Design and Implementation

In order to improve the applicability of the evaluation algorithm (applicable to single-path transmission and multi-path transmission), this section considers converting the routing scheme into a single piece of data and performs real-time evaluation. When a node generates a routing scheme, routing scheme is converted into a single piece of data before transmitting data according to the scheme. To determine whether the data is abnormal is to evaluate in real time whether the routing scheme is feasible.

To improve evaluation accuracy and further reduce evaluation time, we employ machine learning algorithms to evaluate the routing scheme.

Unlabeled samples are more readily available than labeled samples. Since supervised learning requires all training data to be labeled, we chose semi-supervised learning with only a few labeled samples. Since there are only two types of labels, abnormal and non-anomalous, we chose a semi-supervised learning algorithm suitable for binary classification problems. Here, we chose the Semi-Supervised Support Vector Machine (S3VM). This type of algorithm has the following advantages: (1) There are less restrictions on the form of input data, which is conducive to reuse and training data generation. (2) The storage overhead is low, which is beneficial for us to process large-scale data. (3) It is simple and effective. Compared with other semi-supervised learning algorithms, the latency is relatively low, which can properly compensate for the timeliness shortcomings of evaluation methods.

The classic algorithm in the semi-supervised support vector machine is the Transduction Support Vector Machine (TSVM) [11].

**Assumption:** Given a labeled sample set $D_{n}=\left\{\left(x_{1},y_{1}\right),\left(x_{2},y_{2}\right),\ldots ,\left(x_{n},y_{n}\right)\right\}$ and an unlabeled sample set $D_{m}=\left\{x_{n+1},x_{n+2},\ldots ,x_{n+m}\right\}$. $y_{i}\in \left\{-1,1\right\}$ (1 means abnormal, −1 means not abnormal), n≪m.

The goal of TSVM is to predict the label of $D_{m}$ (that is  ^y={^yn+1, ^yn+2,…, ^yn+m}) such that it satisfies Equation (1).

(1) minω,b, ^y,ξ12||ω||22+Cn∑i=1nξi+Cm∑i=n+1n+mξis.t. yi(ωTxi+b)≥1−ξi, i=1,…,n ^yi(ωTxi+b)≥1−ξi, i=n+1,…,n+mξi≥0, i=1,…,n+m

In Equation (1), ξ is the relaxation vector; $C_{n}$ and $C_{m}$ are user-defined parameters for balancing the importance of labeled samples and unlabeled samples; (ω,b) determines a dividing hyperplane. TSVM continuously tries to assign labels to unlabeled samples, and the goal is to determine the partitioning hyperplane that maximizes the separation over all samples.

The flow of TSVM is shown in Algorithm 1. Algorithm 1 obtains the predicted labels for unlabeled samples and a final SVM model (hereinafter referred to as the TSVM model), which can be used to predict new samples.

**Algorithm 1:** TSVM1: Input:  labeled sample set $D_{n}=\left\{\left(x_{1},y_{1}\right),\left(x_{2},y_{2}\right),\ldots ,\left(x_{n},y_{n}\right)\right\}$,2:           unlabeled sample set $D_{m}=\left\{x_{n+1},x_{n+2},\ldots ,x_{n+m}\right\}$,3:           parameters $C_{n}$ and $C_{m}$
4: Initialize $C_{n}$ and $C_{m}$, $C_{n}\gg C_{m}$;5: Train an initial model SVM0 with $D_{n}$;6: Predict the label of $D_{m}$ with SVM0, obtain  ^y={ ^yn+1, ^yn+2,…,^yn+m};7:       While $C_{m}<C_{n}$
8:         Knowing $D_{n}$, $D_{m}$, $C_{n}$, $C_{m}$ and  ^y, obtain (ω,b) and ξ according to formula 1;9:             While ∃{i,j|( ^yi ^yj<0)∧(ξi>0)∧(ξj>0)∧(ξi+ξj>2)}
10:                ^yi = − ^yi;11:                ^yj = − ^yj;12:                Knowing $D_{n}$, $D_{m}$, $C_{n}$, $C_{m}$ and  ^y, obtain (ω,b) and ξ according to formula 1;13:          End while14:          $C_{m}=\min\left\{2C_{m},C_{n}\right\}$;15:      End while16:      Output:   ^y={ ^yn+1, ^yn+2,…, ^yn+m}, TSVM = SVM_final_

We generate the initial multi-path routing scheme according to the wireless sensor network multi-path routing algorithm proposed in reference [4]. The scheme includes p paths sorted according to the selection probability from large to small, and each path must have a node with the smallest remaining energy. The unlabeled samples in this paper contain the energy consumption and remaining energy of these p nodes.

Due to the different training data sets (including labeled samples and unlabeled samples) corresponding to different p and the high cost of manually obtaining training data, it is difficult for us to obtain enough training data for all possible p. To ensure efficient operation of TSVM and avoid overfitting, we need to ensure that there is enough training data. Therefore, we employ data augmentation to improve TSVM in an attempt to effectively augment the training data.

Supervised data augmentation is to expand more labeled data from the original labeled data through some transformation method, and then use the original data and augmented data to jointly train the model. The current general method is to fit the real sample distribution by adding sample points to continuous discrete sample points. We build on this idea for data augmentation in semi-supervised tasks.

We expand positive labeled samples, negative labeled samples and unlabeled samples, respectively. We leave the markup the same and expand in the same way. The expansion method is to insert a sample point $x_{\mathrm{new}}$ into the two discrete sample points $x_{i}$ and $x_{j}$, and the calculation method of the interpolation is as shown in Equation (2).

(2) $$\begin{array}{c} \lambda \sim \mathrm{Beta}\left(\alpha ,\;\beta \right),\alpha ,\beta \in \left(0,1\right)\\ x_{\mathrm{new}}={\lambda *x}_{i}+\left(1-\lambda \right){*x}_{j},\;i,j\in \left(1,p\right) \end{array}$$

In Equation (2), α and β are user-defined parameters, which are generally set to 0.5.

The flow of data enhancement for TSVM is shown in Algorithm 2.

**Algorithm 2:** Data Enhancement for TSVM1: Read the training data set according to the number of paths p involved in the routing scheme;2:       Input: labeled sample set $D_{n}=\left\{\left(x_{1},y_{1}\right),\left(x_{2},y_{2}\right),\ldots ,\left(x_{n},y_{n}\right)\right\}$,3:                 unlabeled sample set $D_{m}=\left\{x_{n+1},x_{n+2},\ldots ,x_{n+m}\right\}$,4:                 parameters $C_{n}$ and $C_{m}$
5:       Initialize $C_{n}$ and $C_{m}$, $C_{n}\gg C_{m}$;6:       // Expand labeled samples7:       Train an initial model SVM0 with $D_{n}$;8:       Split $D_{n}$ into $D_{n+}$ and $D_{n-}$ according to the positive or negative of y, and the number of samples are recorded as n+ and n−9:       Perform high-dimensional clustering on the de-labeled sample sets of $D_{n+}$ and $D_{n-}$;10:       FOR k=1 to n+/10
11:            Randomly select a pair of samples $\left(x_{i},x_{j}\right)$ from the largest cluster of $D_{n+}$;12:            Generate $x_{\mathrm{new}}$ according to Equation (2);13:            Predict the label of $x_{\mathrm{new}}$ with SVM0, obtain $y_{\mathrm{new}}$;14:            IF $y_{\mathrm{new}}=1$
15:                  Merge $\left(x_{\mathrm{new}},y_{\mathrm{new}}\right)$ into $D_{n}$;16:            END IF17:       END FOR18:       FOR k=1 to n−/10
19:            Randomly select a pair of samples $\left(x_{i},x_{j}\right)$ from the largest cluster of $D_{n-}$;20:            Generate $x_{\mathrm{new}}$ according to Equation (2);21:            Predict the label of $x_{\mathrm{new}}$ with SVM0, obtain $y_{\mathrm{new}}$;22:            IF $y_{\mathrm{new}}=-1$
23:                  Merge $\left(x_{\mathrm{new}},y_{\mathrm{new}}\right)$ into $D_{n}$;24:            END IF25:       END FOR26:       // Expand unlabeled samples27:       Train a model TSVM0 with $D_{n}$, $D_{m}$, $C_{n}$, $C_{m}$;28:       Perform high-dimensional clustering on $D_{m}$;29:       FOR k=1 to m/10
30:            Randomly select a pair of samples $\left(x_{i},x_{j}\right)$ from the largest cluster of $D_{m}$;31:              Generate $d_{k}=x_{\mathrm{new}}$ according to Equation (2);32:        END FOR33:          Train a model TSVM1 with $D_{n},{\;D}_{m},{\;C}_{n}$, $C_{m}$;34:          FOR k=1 to m/10
35:              Predict the label of $d_{k}$ with TSVM0 and TSVM1, obtain $y_{\mathrm{new}}^{0}$ and $y_{\mathrm{new}}^{1}$;36:              IF $y_{\mathrm{new}}^{0}=y_{\mathrm{new}}^{1}$
37:                  Merge $d_{k}$ into $D_{m}$;38:              END IF39:          END FOR40:          Output:    Expanded $D_{n}$ and $D_{m}$


It is assumed that the routing scheme involves p paths in total, and each path has a minimum residual energy node, so the scheme includes p minimum residual energy nodes. The energy consumption of the i-th node is ${\mathrm{load}}_{i}$, and the remaining energy is ${\mathrm{free}}_{i}$. The scheme to be checked is $x=\left({\mathrm{load}}_{1},{\mathrm{free}}_{1},\cdots ,{\mathrm{load}}_{p},{\mathrm{free}}_{p}\right)$. The label y equal to 1 indicates that x is abnormal. The equation for determining the label is Equation (3). When the load ratio of a node exceeds a predetermined threshold (which may cause the node to fail) or the variance of the load of all nodes exceeds a predetermined threshold (indicating that the energy distribution is unreasonable, which may eventually lead to node failure), the logical value of Equation (3) is 1, that is, x is abnormal.

(3) $$\left(\exists \left\{i|\frac{{\mathrm{load}}_{i}}{{\mathrm{free}}_{i}}>\alpha \right\}\right)\vee (D\left(\left\{{\mathrm{load}}_{1},\cdots ,{\mathrm{load}}_{p}\right\}\right)>\beta )$$

Combining Algorithms 1 and 2, we design and implement the real-time evaluation algorithm based on semi-supervised learning (RESL), whose flow is as in Algorithm 3.
**Algorithm 3:** Real-time evaluation algorithm based on semi-supervised learning (RESL)1:  Read the training data set according to the number of paths p involved in the routing scheme;2:  Input: labeled sample set $D_{n}=\left\{\left(x_{1},y_{1}\right),\left(x_{2},y_{2}\right),\ldots ,\left(x_{n},y_{n}\right)\right\}$, 3:             unlabeled sample set $D_{m}=\left\{x_{n+1},x_{n+2},\ldots ,x_{n+m}\right\}$,4:             the scheme to be checked x
5:  IF (n<100)⋁(m<400)
6:        Determine whether x is abnormal according to Equation (3), obtain y;7:  ELSE8:      IF (n<200)⋁(m<800)
9:             Perform Algorithm 2;10:             Perform Algorithm 1, obtain TSVM;11:             Predict x with TSVM, obtain y;12:         ELSE13:             Perform Algorithm 1;14:             Predict x with TSVM, obtain y;15:         END IF16:  END IF17:  Output: y

When the training data is not enough to train an effective model, use formula (3) to obtain the label; when the training data is enough to train the model but not enough to train a better model, the label is obtained after the data is enhanced; when the training data is enough to train a good model, the label is obtained directly.

### 2.2. Comparative Experiment

Taking time and accuracy as evaluation indicators, we compared the evaluation algorithm (RESL) proposed in this paper with the evaluation algorithm proposed in references [9,10]. The fuzzy evaluation algorithm (FEA) proposed in reference [9] has a very low evaluation time, but the evaluation accuracy is not high; the adaptive evaluation algorithm (AEA) proposed in reference [10] has a high evaluation accuracy, but the evaluation time is not low.

We measured and recorded the evaluation time and evaluation accuracy of the three algorithms for the same multi-path routing scheme.

1. Evaluation accuracy

We conducted a total of 500 trials and the results are shown in Table 1. We used three algorithms to measure each of the 50 sets of data corresponding to p = 1~10 and recorded the evaluation accuracy.

If the path is selected more than twice (that is, the top two paths are not feasible), the actual result of the routing scheme is abnormal. We converted the routing plan to the corresponding data to be checked and used three evaluation algorithms to evaluate the data to be checked to obtain the judgment result (whether it is abnormal). If the judgment result is the same as the actual result, the evaluation is regarded as accurate. Since FEA and AEA both evaluate the multi-path routing scheme multiple times, if at least one abnormality occurs in the multiple evaluations, the judgment result is abnormal.

**Figure 2:**
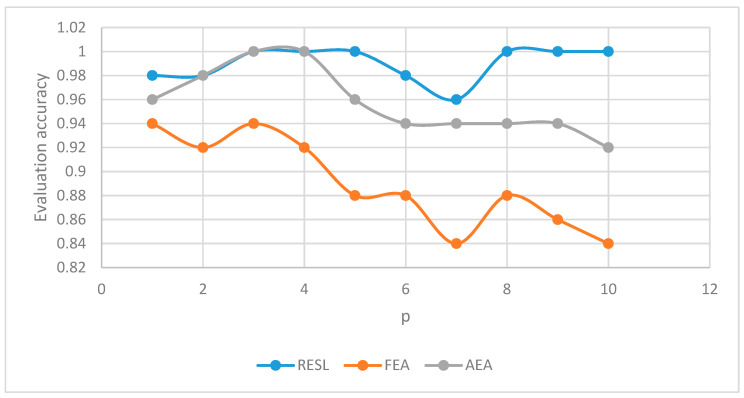
Evaluation accuracy.

Plot Table 1 as Figure 2. It can be seen from Figure 2 that the evaluation accuracy of RESL is much higher than that of FEA and slightly higher than that of AEA.

2. Evaluation time

We conducted a total of 150 trials and the results are shown in Table 2. We used three algorithms to measure each of the 50 sets of data corresponding to p = 1~10 and recorded the running time of the evaluation algorithm.

Since both FEA and AEA are single-path evaluations, they need to perform multiple evaluations for the multi-path solution, so their evaluation time counts the total time of multiple evaluations.

**Figure 3:**
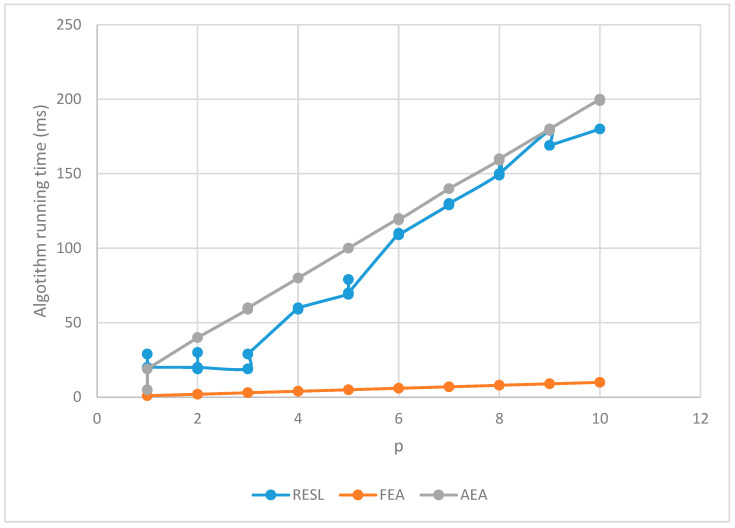
Algorithm running time.

Plot Table 2 as Figure 3. It can be seen from Figure 3 that the algorithm running time of RESL is lower than AEA but higher than FEA.

Although FEA has a great advantage in algorithm running time, its evaluation accuracy is not high. The time cost of evaluating errors introduced (rerouting time, etc.) is much higher than the running time of evaluating algorithms. Therefore, the overall evaluation time of using FEA in an evaluation-based routing algorithm is unstable and extremely time-consuming.

**Assumption** **1:**  *Given that the running time of the algorithm is t, the evaluation accuracy is c and the time introduced by the evaluation error is f.*

The evaluation time T for evaluating the routing scheme is defined as Equation (4).

(4) T=c∗t+(1−c)∗f

**Figure 4:**
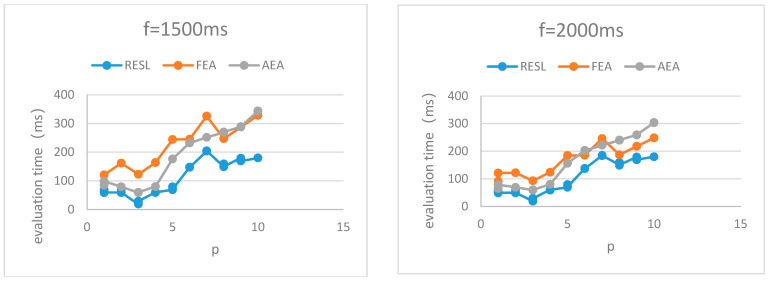
Evaluation time.

Given different f, we combine Table 1 and Table 2 to obtain the evaluation time of the three algorithms, as shown in Figure 4.

It can be seen from Figure 4 that the evaluation time of RESL is lower than that of FEA and AEA.

In conclusion, RESL outperforms the two comparison algorithms in evaluation time and evaluation accuracy. (1) Compared with FEA with extremely low algorithm running time, the evaluation accuracy of RESL is much higher than that of FEA. Although the algorithm running time of RESL is higher than that of FEA, the evaluation time is lower than that of FEA. (2) Compared with AEA, which has a higher evaluation accuracy, the evaluation accuracy of RESL is slightly higher than that of AEA and the evaluation time is lower than that of AEA.

## 3. Multi-Path Routing Algorithm for Wireless Sensor Network Based on 

### 3.1. Semi-Supervised Learning (MRSSL)

In this section, we design and implement a semi-supervised learning-based multi-path routing algorithm for wireless sensor networks (MRSSL). We prove that the algorithm has significant effect in improving the accuracy of the routing scheme through comparative experiments.

### 3.2. Algorithm Design and Implementation

The basic idea of MRSSL is to use the RESL proposed in Section 2 (i.e., Algorithm 3) to evaluate the multi-path routing scheme, and then adjust the scheme that evaluates abnormally (i.e., y = 1) until the evaluation is normal (i.e., y = 0) or end of iteration.

According to this idea, we designed and implemented the multi-path routing algorithm for wireless sensor networks based on semi-supervised learning (MRSSL), whose flow is shown in Algorithm 4.
**Algorithm 4:** Multi-path routing algorithm for wireless sensor networks based on semi-supervised learning (MRSSL)
1:  Generate the initial multi-path routing scheme x according to the wireless sensor network multi-path routing algorithm proposed in reference [4];2:  Read the training data set according to the number of paths p involved in the routing scheme;3:  Input: labeled sample set $D_{n}=\left\{\left(x_{1},y_{1}\right),\left(x_{2},y_{2}\right),\ldots ,\left(x_{n},y_{n}\right)\right\}$, 4:            unlabeled sample set $D_{m}=\left\{x_{n+1},x_{n+2},\ldots ,x_{n+m}\right\}$,5:            parameter N6:  FOR n = 1 to N7:        Perform Algotithm 3, obtain y;8:        IF y=0
9:              break out;10:      Else11:            make an adjustment to x;12:      END IF13:  END FOR14:  Output: final x

The initial scheme in Algorithm 4 is $x=\left({\mathrm{load}}_{1},\;{\mathrm{free}}_{1},\cdots ,{\mathrm{load}}_{p},\;{\mathrm{free}}_{p}\right)$. Every time the scheme is adjusted, the value pairs are reordered (${\mathrm{load}}_{i},\;{\mathrm{free}}_{i}$). The abnormality of the initial scheme indicates that the top two paths in the scheme are infeasible, so the constraint condition of the adjustment scheme is to adjust the top two value pairs of the initial scheme to the end.

The output of Algorithm 4 is the final multi-path routing scheme. The multiple paths in the routing scheme are ordered from high to low probability of selection.

### 3.3. Comparative Experiment

In order to verify the advantages of MRSSL in improving the accuracy of multi-path routing schemes, we compared it with two classical multi-path routing algorithms (CEMRM proposed in [4] and RMBDP proposed in [5]).

**Figure 5:**
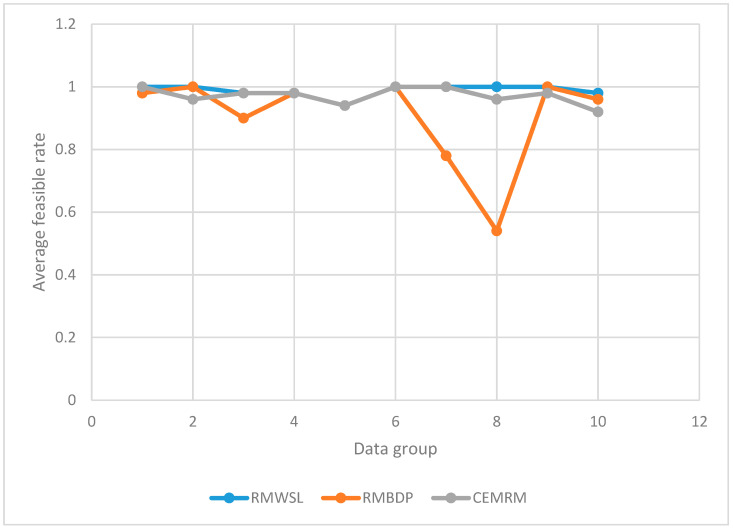
Average feasible rate.

We tested the routing schemes generated by three multi-path routing algorithms. In each test, the existence of feasible paths in the top two paths in the routing scheme indicates that the scheme is feasible. We grouped 50 different tests as a set and recorded the average feasibility rate. We conducted ten groups of tests, and the experimental results are shown in Table 3 (corresponding to Figure 5). The higher the average feasible rate of the routing scheme, the higher the accuracy of the routing scheme.

Executing the same number of schemes multiple times, a higher scheme feasibility rate indicates that there is a higher probability of including a feasible scheme, indicating that the scheme accuracy is higher.

It can be seen from Figure 5 that the average feasibility rate of the scheme generated by MRSSL is higher than that of the two comparison methods, indicating that MRSSL has advantages in improving the accuracy of the multi-path routing scheme.

## 4. Conclusions

Multi-path transmission can well solve the problems caused by single-path transmission. However, existing multi-path routing algorithms make it difficult to ensure the accuracy of the generated routing scheme. The innovation of this paper is to solve the problem of low accuracy of multi-path routing schemes in wireless sensor networks (WSN) by means of evaluation. First, we designed and implemented the real-time evaluation algorithm based on semi-supervised learning (RESL). We proved that RESL is better in evaluation time and evaluation accuracy through comparative experiments. Then, we combined RESL to design and implement the multi-path routing algorithm for wireless sensor networks based on semi-supervised learning (MRSSL). We proved that MRSSL has advantages in improving the accuracy of the multi-path routing scheme through comparative experiments.

The method proposed in this paper can be well applied to the routing of WSN. In the research process, there are still shortcomings: (1) Due to the limited training data and time-consuming acquisition, the evaluation method proposed in this paper can only be a semi-supervised algorithm with lower evaluation accuracy. Later, if a lot of time can be used to further improve the training data, it can be replaced with a supervised algorithm with a higher evaluation accuracy. (2) Whether the method proposed in this paper is applicable to other network areas should be explored in the next work.

## Figures and Tables

**Table 1:** Evaluation accuracy.

The Number of Paths	Evaluation Accuracy		
	RESL	FEA	AEA
p = 1	0.98	0.94	0.96
p = 2	0.98	0.92	0.98
p = 3	1	0.94	1
p = 4	1	0.92	1
p = 5	1	0.88	0.96
p = 6	0.98	0.88	0.94
p = 7	0.96	0.84	0.94
p = 8	1	0.88	0.94
p = 9	1	0.86	0.94
p = 10	1	0.84	0.92

**Table 2:** Algorithm running time.

The Number of Paths	Algorithm Running Time (ms)		
	RESL	FEA	AEA
p = 1	19, 29, 19, 19, 20	1, 1, 1, 1, 1	5, 19, 19, 19, 19
p = 2	20, 20, 30, 19, 20	2, 2, 2, 2, 2	40, 40, 40, 40, 40
p = 3	19, 29, 29, 29, 29	3, 3, 3, 3, 3	59, 60, 59, 60, 60
p = 4	60, 60, 59, 59, 60	4, 4, 4, 4, 4	80, 80, 80, 80, 80
p = 5	69, 69, 79, 69, 70	5, 5, 5, 5, 5	100, 100, 100, 100, 100
p = 6	110, 109, 109, 109, 109	6, 6, 6, 6, 6	120, 119, 120, 120, 119
p = 7	129, 129, 129, 130, 130	7, 7, 7, 7, 7	140, 140, 140, 140, 140
p = 8	150, 159, 149, 150, 150	8, 8, 8, 8, 8	159, 160, 160, 160, 160
p = 9	179, 169, 169, 169, 169	9, 9, 9,9, 9	180, 180, 180, 179, 180
p = 10	180, 180, 180, 180, 180	10, 10, 10, 10, 10	200, 200, 199, 199, 200

**Table 3:** Average feasible rate.

Data Group	Average Feasible Rate		
	RMWSL	RMBDP	CEMRM
1	1	0.98	1
2	1	1	0.96
3	0.98	0.90	0.98
4	0.98	0.98	0.98
5	0.94	0.94	0.94
6	1	1	1
7	1	0.78	1
8	1	0.54	0.96
9	1	1	0.98
10	0.98	0.96	0.92

## Data Availability

Data is contained within the article. The data presented in this study are available in article.

## References

[1] Sun L.M. (2015). Wireless Sensor Network.

[2] Moharamkhani E., Zadmehr B., Memarian S., Saber M.J., Shokouhifar M. (2021). Multiobjective fuzzy knowledge-based bacterial foraging optimization for congestion control in clustered wireless sensor networks. Int. J. Commun. Syst..

[3] Esmaeili H., Hakami V., Minaei B., Shokouhifar M. (2022). Application-specific clustering in wireless sensor networks using combined fuzzy firefly algorithm and random forest. Exp. Syst. Appl..

[4] Rahul C.S., Jan M.R. Energy Aware Routing for Low Energy Ad Hoc Sensor Networks. Proceedings of the IEEE Wireless Communications and Networking Conference.

[5] Wang Y.X., Nie G.F., Tian H. A Graph Routing Algorithm Enhancing Wireless Sensor Networks Lifetime. Proceedings of the 2021 International Conference on Space-Air-Ground Computing (SAGC).

[6] Addisalem G., Lobiyal D.K., Jemal H.A. (2019). Energy Efficient Multipath Routing Algorithm for Wireless Multimedia Sensor Network. Sensors.

[7] Priti M., Kapil G. (2021). Energy balanced, delay aware multi-path routing using particle swarm optimisation in wireless sensor networks. Sens. Netw..

[8] Messous S., Liouane N. Multi-hop energy-efficient routing protocol based on Minimum Spanning Tree for anisotropic Wireless Sensor Networks. Proceedings of the 2019 International Conference on Advanced Systems and Emergent Technologies.

[9] Li J., Chang X., Ren Y. An effective path load balancing mechanism based on SDN. Proceedings of the 13th International Conference on Trust, Security and Privacy in Computing and Communications.

[10] Lee K.M., Teng W.G., Hou T.W. (2016). DRASE: A Dynamic Rescheduling and Self-Adaptive Estimation Technique to Enhance ACS Throughputs in CWMP. IEEE Commun. Lett..

[11] Zhou Z.H. (2018). Machine Learning.

